# Neutrophil Extracellular DNA Traps in Response to Infection or Inflammation, and the Roles of Platelet Interactions

**DOI:** 10.3390/ijms25053025

**Published:** 2024-03-05

**Authors:** William A. Chen, Danilo S. Boskovic

**Affiliations:** 1 Division of Biochemistry, Department of Basic Sciences, School of Medicine, Loma Linda University, Loma Linda, CA 92350, USA; wachen@llu.edu; 2 Department of Pharmaceutical and Administrative Sciences, School of Pharmacy, Loma Linda University, Loma Linda, CA 92350, USA; 3 Department of Earth and Biological Sciences, School of Medicine, Loma Linda University, Loma Linda, CA 92350, USA

**Keywords:** coagulation, neutrophils, NETs, platelets, thrombosis, immunity

## Abstract

Neutrophils present the host’s first line of defense against bacterial infections. These immune effector cells are mobilized rapidly to destroy invading pathogens by (a) reactive oxygen species (ROS)-mediated oxidative bursts and (b) via phagocytosis. In addition, their antimicrobial service is capped via a distinct cell death mechanism, by the release of their own decondensed nuclear DNA, supplemented with a variety of embedded proteins and enzymes. The extracellular DNA meshwork ensnares the pathogenic bacteria and neutralizes them. Such neutrophil extracellular DNA traps (NETs) have the potential to trigger a hemostatic response to pathogenic infections. The web-like chromatin serves as a prothrombotic scaffold for platelet adhesion and activation. What is less obvious is that platelets can also be involved during the initial release of NETs, forming heterotypic interactions with neutrophils and facilitating their responses to pathogens. Together, the platelet and neutrophil responses can effectively localize an infection until it is cleared. However, not all microbial infections are easily cleared. Certain pathogenic organisms may trigger dysregulated platelet–neutrophil interactions, with a potential to subsequently propagate thromboinflammatory processes. These may also include the release of some NETs. Therefore, in order to make rational intervention easier, further elucidation of platelet, neutrophil, and pathogen interactions is still needed.

## 1. Introduction

Granulopoiesis is the process by which hematopoietic stem cells (HSCs) differentiate into mature neutrophils [1]. Self-renewing HSCs, produced in bone marrow, first give rise to multipotent progenitors (MPPs). While MPPs are incapable of self-renewal, they still retain the ability to differentiate into a variety of mature blood cells [2]. MPPs can yield oligopotent common myeloid progenitors, which go on to generate granulocyte-monocyte progenitors (GMPs). Myeloblasts, which are derived from lineage restricted GMPs, serve as the first precursor cells which are terminally committed to the neutrophil cell line [3]. During differentiation, they undergo a series of maturation steps that are regulated by growth factors (granulocyte–colony-stimulating factor (G-CSF) and granulocyte–macrophage colony-stimulating factor (GM-CSF)), transcription factors (cytosine-cytosine-adenosine-adenosine-thymidine-enhancer-binding protein (C/EBP) and runt-related transcription factor 1 (RUNX1)), as well as cytokine signaling molecules (interleukins IL-3, IL-17A, and IL-23) [4].

Classical models suggests that the neutrophils are primarily derived from myeloid lineage cells. However, nonclassical progenitor cells with lymphoid and myeloid differentiation potential, designated as lymphoid-primed MPPs, may also develop into neutrophils [5,6]. The priming of multiple gene expression programs supports lineage promiscuity [7]. By remaining uncommitted to one cell line, the hematopoietic system is capable of adapting to changes or fluctuations within the host microenvironment [8]. Then, in response to exogenous inflammatory factors, HSCs can commit progenitor cells towards the needed appropriate cell type. This enables the rapid population replenishment of short-lived blood cells, such as neutrophils, as they are depleted during host immune responses to bacterial infections.

### 1.1. Neutrophil Granules

Pathogenic infections or endothelial damage can trigger an immune response by releasing chemokines to guide activated leukocytes toward a target site. Neutrophils account for 50–70% of all circulating leukocytes within the host [9]. These immune effector cells present the first line of defense against infections, restricting the dissemination of pathogenic bacteria and neutralizing them. The safe transport of cytotoxic antimicrobial enzymes to the infection site is crucial for a competent neutrophil response.

Secretory granules are formed during neutrophil differentiation. They are first observed between the transition from the myeloblast to the promyelocyte stage [10]. The following four main types of granules are recognized: (a) azurophilic, (b) specific, (c) gelatinase, and (d) secretory vesicles [11]. Within these densely packed granules are antimicrobial proteins that contribute toward functional neutrophil antibacterial responses. Azurophilic granules contain myeloperoxidases (MPOs), defensins, and serine proteases, including neutrophil elastase (NE) and cathepsin G [12]. Specific granules carry lactoferrin, collagenase, and nicotinamide adenine dinucleotide phosphate (NADPH) oxidase, while gelatinase granules contain matrix metalloproteases (MMPs), such as gelatinase and leukolysin [13,14]. The secretory vesicles predominantly contain plasma-derived proteins, such as human serum albumin, but the vesicle membranes carry β_2_ integrins (CD11b/CD18) and FcγIII receptors (CD16) [15,16]. Additionally, lysozymes are observed in all four types of granules [17].

### 1.2. Neutrophil Antimicrobial Mechanisms

Two antimicrobial mechanisms are characteristic of neutrophils. First, recruited neutrophils can phagocytose any encountered bacteria, or derived material, for elimination using an NADPH oxidase-dependent mechanism. The NADPH oxidase catalyzes the reduction of oxygen to form several reactive oxygen species (ROS) that are subsequently released into phagosomes via oxidative bursts [18]. Intracellular degranulation enhances the antimicrobial function of phagocytosis. Following the ingestion of pathogens, mobilized granules fuse with the phagosomes, releasing their antimicrobial enzymes into the phagosomal lumen [19]. These proteins work together with ROS to digest trapped pathogens. Second, the activation of neutrophils can induce the degranulation and release of bactericidal proteins into the local environment to facilitate bacterial clearance [20]. The activation of the secretory vesicles is easiest, followed by that of gelatinase, specific, and lastly azurophilic granules [21].

### 1.3. Neutrophil Adhesion and Extravasation

In proximity to bacterial infection, or in response to vascular damage, endothelial cells produce chemokines to form a chemoattractant gradient, guiding circulating neutrophils towards the affected site. The mobilized neutrophils adhere to the endothelium in a multistep mechanism, involving capture, rolling, arrest, crawling, and transmigration. Pathogenic or inflammatory stimuli activate the endothelial cells and trigger the adhesion cascade. Proinflammatory cytokines induce E-selectin and P-selectin expression in endothelial cells [22]. E-selectin is synthesized de novo, while P-selectin is released from Weibel–Palade bodies (WPBs) located within endothelial cells [23]. Both selectins are cell adhesion molecules (CAMs), mediating interactions between neutrophils and endothelium, with a central role in the capture of neutrophil from circulation [24]. Following the capture, neutrophils transition into a period of slow rolling, while translocating along the vascular wall. The upregulation of neutrophil L- and P-selectin expression supports neutrophil catch–slip bonds [25]. A biphasic relationship between the shear stress forces characterizes these catch–slip interactions. Initially, characteristic of catch bonds, the lifetimes of the bonds increase with force. Following a maximum, however, the lifetimes decrease with force, indicative of slip bonds [26]. Consequently, these catch–slip bonds are flow-dependent interactions functioning at specific shear stresses [27]. At low shear stress, the movement of neutrophils is erratic, largely due to transient interactions. Under higher shear stress, the movement of rolling neutrophils tends to be more stable with slower detachments.

Alternating between catch–slip bonds promotes a slow rolling motion to reduce the velocity of neutrophils [28]. Rolling neutrophils can also interact with immobilized proinflammatory chemokines, including chemokine (C-X-C motif) ligand (CXCL) 1, CXCL12, or IL-8, on the inflamed endothelium, triggering signaling pathways that activate neutrophil-expressed leukocyte function-associated antigen-1 (LFA-1, CD11a/CD18) and macrophage-1 antigen (MAC-1, CD11b/CD18) receptors [29]. Activated β_2_-integrins switch into an open conformation and bind to endothelial intercellular cell adhesion molecule (ICAM)-1 and ICAM-2 with high affinity [30,31]. MAC-1 facilitates crawling along the endothelial wall, while the sequential binding of LFA-1 and MAC-1 to ICAM-1 mediates firm adhesion and stabilizes receptor–ligand interactions, respectively [32,33]. LFA-1 and MAC-1 interactions with ICAM-2 stabilize neutrophil rolling under high shear stress, via tether-based slings that envelop neutrophils as well as mediate neutrophil transmigration through the endothelial barrier [34,35].

Following firm adhesion, neutrophils locate permissive sites to penetrate through the endothelial barrier entering into local tissue. During this extravasation process, neutrophils undergo cell polarization with morphological changes to form a leading edge (pseudopod) and a trailing end (uropod) [36]. Using a MAC-1-dependent mechanism, neutrophils extend out lamellar-like pseudopodia to crawl towards suitable areas and to position themselves for infiltration [33]. The rapid assembly of actin filaments along the protruding leading edge creates densely branched actin networks that drive the crawling movement along the vascular wall, while also anchoring neutrophils to the surface and maintaining adhesion [37]. The formation of uropods serves to concentrate contractile force in the rear area [38]. Uropods redistribute the generated traction stress towards the frontward pseudopods, allowing the forward movement of neutrophils in the direction of the chemotactic signal while searching for permissive sites for extravasation [39].

The transmigration of neutrophils through the endothelial barrier occurs via the two following characterized mechanisms: paracellular and transcellular. The paracellular pathway of diapedesis involves neutrophils crawling to cell–cell junctions and mediating the disassembly of vascular endothelial–cadherin complexes to create a transient opening between endothelial cells [40]. The movement through the gap is a highly regulated process, with neutrophil adhesion to the endothelial surface triggering signaling pathways that mediates cell–cell interactions by increasing intracellular calcium levels, remodeling actin cytoskeleton, and activating Src tyrosine kinase and Ras homolog family member A (RhoA) guanosine triphosphatase (GTPase) [41]. Transcellular neutrophil migration involves the formation of a channel that spans the entirety of the endothelial cell. To cross the cellular barrier, neutrophils use pseudopods to force invaginations into the endothelial cells and remodel the membrane by disrupting actin filaments and microtubules [42]. As they invade past the endothelial membrane surface to form a transcellular pore, neutrophils fuse their membrane to the endothelium in a soluble N-ethylmaleimide-sensitive factor attachment protein receptor (SNARE)-dependent manner [43]. The transmigration mechanism, for both the paracellular and transcellular passages from circulation into interstitial tissue, involves cell–cell interactions between neutrophil and endothelial CAMs and receptors including CD99, CD99L2, ICAM-1, ICAM-2, junctional adhesion molecule (JAM)-A, JAM-C, platelet endothelial cell adhesion molecule (PECAM)-1, VCAM-1, LFA-1, and MAC-1 [44]. The paracellular pathway appears to be the preferred mechanism [45]. However, it is unclear how the selection between the paracellular and transcellular pathways is made. Contributing factors may include endothelial junctional tightness, endothelial integrin expression density, cellular biomechanical properties such as cytoskeletal or matrix rigidity, leukocyte subtypes, and inflammatory signal strength [46,47,48,49,50,51,52].

## 2. Neutrophil Extracellular DNA Traps

**Figure 1:**
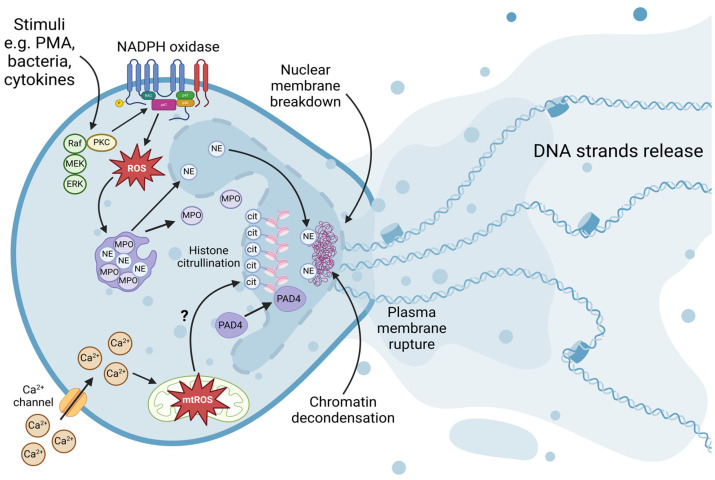
Formation of neutrophil extracellular DNA traps. Two mechanisms of NETs formation include a NOX-dependent and a NOX-independent pathway. Stimuli triggering the NOX-dependent pathway result in the activation of the Raf/MEK/ERK pathway, the activation of NADPH oxidase, and the production of cytosolic ROS. The NOX-independent pathway can be activated via calcium ionophores, and leads to the production of mitochondrial ROS. The role of mitochondrial ROS in PAD4-mediated histone citrullination or NE- and MPO-mediated chromatin decondensation is still under investigation. Downstream effects of these activated pathways can include the breakdown of the nuclear membrane, PAD4-mediated histone citrullination, NE- and MPO-mediated chromatin decondensation, the rupture of the plasma membrane, and the extracellular release of DNA strands. Created with BioRender.com.

A specific form of leukocyte antimicrobial functions is expressed via the formation of neutrophil extracellular DNA traps (NETs). In response to pathogenic infection or proinflammatory stimuli, the decondensed chromatin fibers from neutrophils are released into the extracellular space [53,54]. Embedded within the chromatin network are histones and antimicrobial proteins, including MPOs, NEs, cathepsin G, and proteinase 3 [55,56,57,58]. NETs are web-like structures that immobilize and neutralize pathogens by exposing them to a high concentration of localized antimicrobial proteins (Figure 1). The release mechanism for NETs appears to include the hypercitrullination of histones by peptidylarginine deiminase 4 (PAD4), the MPO- and NE-mediated decondensation of neutrophil chromatin, the breakdown of nuclear and plasma membranes, and chromatin release [59,60,61].

### 2.1. Formation of NETs

#### 2.1.1. Peptidylarginine Deiminase 4-Mediated Hypercitrullination

PAD4 is a calcium-dependent enzyme primarily localized in the nucleus and in neutrophil granules [62]. It mediates histone deimination, a post-translational modification that converts arginine residues into citrulline throughout the histone protein structures [63]. However, the majority are clustered within the N-terminus tail regions [64]. This histone tail stabilizes higher order chromatin structures by mediating DNA–histone (DNA folding) and nucleosome–nucleosome (chromatin folding) interactions [65,66]. Furthermore, histone post-translational modifications impact the regulation of chromatin structure and stability [67]. In this context, PAD4 is a contributing factor to chromatin decondensation during NETs formation. The time-dependent citrullination of histone 3 (H3) is observed in HL-60 granulocytes, following stimulation with a calcium ionophore, lipopolysaccharide (LPS), or the recombinant human tumor necrosis factor (TNF) [68]. Conversely, the inhibition of PAD4 activity decreases histone citrullination and chromatin decondensation in HL-60 granulocytes [61]. Furthermore, H3 citrullination, chromatin decondensation, and NET formation are not observed in neutrophils isolated from PAD4 knockout mice [69]. Since arginine residues carry a positive charge while citrulline is generally neutral, PAD4-mediated citrullination leads to a net loss of histone positive charges [70]. Consequently, electrostatic interactions between DNA and histones weaken, disrupting higher-order chromatin structures, causing negatively charged DNA strands to unfold from nucleosomes, resulting in chromatin decondensation [71].

#### 2.1.2. Neutrophil Elastase and Myeloperoxidase-Mediated Chromatin Decondensation

Neutrophil elastase (NE) is a serine protease capable of degrading exposed extracellular matrix (ECM) proteins with a specificity for aliphatic amino acids (AA) [72]. It possesses some antimicrobial functions, synergistically eliminating phagocytosed microbes, even in the presence of ROS produced by NADPH oxidase [73]. Myeloperoxidase (MPO) catalyzes the oxidation of chloride anions via a hydrogen peroxide-dependent mechanism [74]. It subsequently produces hypochlorous acid, a cytotoxic reactive oxidant with bactericidal properties [75].

Neutrophil chromatin decondensation is at least in part NE-dependent [60]. In human neutrophil lysates, chromatin decondensation is significantly reduced after treatment with an NE inhibitor, while treatment with an MPO inhibitor does not elicit the same effect. The NE-mediated degradation of H1 followed by that of H4 was time dependent, with chromatin decondensation following a similar pattern [60]. In contrast, H2A, H2B, and H3 were observed to be only partially cleaved when incubated with NE [60]. A similar addition of MPO, however, does not significantly impact the degradation of histones. Meanwhile, pretreating permeabilized nuclei with anti-histone antibodies followed by NE incubation significantly reduced NE-mediated chromatin decondensation, further emphasizing the role of NE. In this context, the H1 linker histone is a key component of higher-order chromatin structure [76]. It regulates the position of DNA binding to the nucleosome core particle and facilitates nucleosome array folding, both of which contribute to chromatin compaction [77]. Because of this, the initial degradation of H1 would potentially weaken DNA–histone interactions, providing NE additional access to nucleosomal proteins, leading to their cleavage and further chromatin unfolding.

By itself, MPO does not impact histone degradation or chromatin decondensation. However, NE-mediated chromatin decondensation is enhanced in the presence of MPO in a concentration-dependent manner. MPO is implicated as a contributing factor for NE translocation into the neutrophil nucleus [78]. Neutrophils isolated from patients with chronic granulomatous disease (CGD) or from donors who are deficient in MPO function have impaired localization of NE into the nucleus, following stimulation with either *Candida albicans* or phorbol 12-myristate 13-acetate (PMA). Instead, NE remains localized within neutrophil granules [78]. Patients with CGD are characterized by a genetic mutation that impairs NADPH oxidase activity and ROS production [79]. Treating isolated normal human neutrophils with PEG-catalase, an enzyme that consumes H_2_O_2_, prior to PMA stimulation inhibits NE release, confirming the contributing role of the oxidative burst [78].

Interestingly, NE colocalizes with actin-based filopods in *C. albicans*-stimulated neutrophils if they are also treated with an NE inhibitor [78]. Additionally, following their activation by *C. albicans*, the actin levels are decreased in a time-dependent manner in whole cell extracts of NETs releasing neutrophils. Finally, the incubation of purified NE with F-actin generates actin fragments, thus supporting the potential role of NE in the cytoskeletal actin reorganization during chromatin decondensation.

Taken together, stimulated neutrophils generate H_2_O_2_ via oxidative bursts, which mediates the NE release from granules in an MPO-dependent manner. NE can degrade cellular actin and enter into the nucleus to cleave histones, promoting chromatin decondensation. In this context, MPO enhances NE release from granules and its translocation into the nucleus to facilitate chromatin decondensation and the release of NETs [60,78].

#### 2.1.3. Nuclear Envelope Breakdown

During NET formation, for chromatin release to be possible, the nuclear envelope and plasma membrane need to be broken down. The permeabilization of the neutrophil plasma membrane occurs after stimulation with purified LPS from *Escherichia coli* [80]. Similarly, in PMA-activated neutrophils isolated from healthy donors, a time-dependent rupturing of the nuclear envelope and plasma membrane is observed [59]. In contrast, such morphological membrane changes, upon PMA stimulation, do not occur in neutrophils isolated from CGD patients [59].

Neutrophils from wild-type mice, infected with *S. aureus*, demonstrate phenotypic stages of their cell nuclei as follows: normal, diffuse, and anuclear [81]. Normal neutrophils have chromatin contained entirely within their nuclei. Diffuse neutrophils exhibit nuclear envelope disruption, suggesting they are undergoing NET formation. Anuclear neutrophils lack intact nuclei and are without intracellular chromatin, consistent with the completed release of NETs. The formation of NETs, therefore, compromises the integrity of the neutrophil nuclear envelope and plasma membranes through as-yet poorly understood mechanisms, including specific roles for lamins from nuclear lamina and cytoskeletal actin, respectively.

Lamins are key structural constituents localized within the nuclear lamina, a dense meshwork of intermediate filaments reinforcing the nuclear envelope [82]. Lamins maintain nuclear architecture by (a) stabilizing its mechanical properties, (b) regulating chromatin organization, and (c) mediating DNA repair [83,84,85]. From this perspective, the disassembly of lamins could play a role in the breakdown of the nuclear envelope during the formation of NETs. The staining of lamin B1 surrounds Hoechst-stained chromatin in PMA-activated and unstimulated isolated human neutrophils [86]. This is consistent with lamin serving as a structural component of the nuclear envelope [86]. After the PMA-activation of neutrophils, NETs are released following lamin B1 degradation, and lamin B1 fragments become observable in cytoplasm. In contrast, lamin B1 staining remains intact without any rupture of the nuclear envelope in unstimulated neutrophils.

The role of lamin in nuclear membrane integrity is regulated at least in part by PAD4, protein kinase C (PKC), and cyclin-dependent kinase (CDK). PAD4 is a contributing factor to the rupture of nuclear lamina [87]. Human neutrophils deficient in PAD4 have reduced lamin B/nuclear envelope rupture, following ionomycin stimulation [87]. PAD4 re-expression in PAD4-deficient neutrophils abolishes this effect, as the disassembly of lamin B and the release of NETs are observed.

Similarly, PKC-mediated phosphorylation is believed to be involved in lamin B disassembly and the breakdown of the nuclear membrane [88]. The nuclear accumulation of PKCα is time dependent in PMA-stimulated human neutrophils. Furthermore, phosphorylated lamin B is measured in isolated human neutrophils activated with PMA. However, treating activated neutrophils with a PKC inhibitor attenuates lamin B phosphorylation and significantly decreases NET formation [88]. It may also be helpful to consider that nuclear envelope rupture, observed during NET production, is at least partially analogous to that occurring during mitosis. Progression through mitosis is known to involve lamin B1 disassembly [89]. The treatment of HeLa cells with CDK1 and PKC inhibitors delays lamin B disassembly, and this prolongs the duration of mitotic progression [89]. Similarly, RNA interference mediating depletion of PKCα also elicits similar effects [89]. Thus, nuclear membrane breakdown during mitosis is dependent on PKC-mediated phosphorylation and the disassembly of lamin B.

In this context, the upregulation of CDK4/6 expression and the time-dependent phosphorylation of lamin A/C is also observed in PMA-activated neutrophils [90]. Conversely, if CDK is inhibited, then NET release is impaired. This can be demonstrated by treating isolated neutrophils with a peptide mimicking p21 [90], a known CDK inhibitor [91]. CDK4/6 activation in quiescent cells plays a role in mitogenic signaling and cell cycle progression, facilitating the transition from G1 to S phase [92]. Lamin A/C phosphorylation appears to weaken the nuclear structural integrity, thus facilitating membrane degradation during mitosis [93]. Altogether, lamins are implicated in processes associated with the breakdown of the nuclear envelope during cell division and during NET formation. Detailed mechanistic steps, however, still need to be elucidated.

#### 2.1.4. Plasma Membrane Rupture

The opening up of neutrophil cell membranes during NET release includes a disruption of the actin cytoskeletal structures, and at least some steps involved in the formation of transmembrane pores. Actin disassembly occurs when NETs are released from neutrophils activated with PMA, while treatment with jasplakinolide, a protein that induces actin polymerization and stabilizes actin filaments, abolished this effect [86]. Yet, preincubating neutrophils with cytochalasin D, an actin polymerization inhibitor, prior to LPS treatment significantly reduces NET release [94]. Interestingly, the cytochalasin D-pretreated neutrophils appear enlarged, with an intact plasma membrane, and cytoplasmic mixing of chromatin. This suggests that actin filament disassembly is an active process in the disruption of the plasma membrane during NET formation.

The formation of transmembrane pores to permeabilize the plasma membrane involves another cytosolic protein, gasdermin D (GDD). Such membrane permeabilization triggers pyroptosis, which represents a distinct type of programmed cell death that occurs under inflammatory conditions [95]. GDD is thought to play a role in NET formation because of its ability to disrupt plasma membrane integrity. Robust NET formation is observed when neutrophils purified from murine bone marrow are primed with Pam3-Cys-Ser-Lys4 (Pam3CSK4) and treated with LPS [96]. However, NET release is attenuated in neutrophils from GDD knockout mice under the same treatment conditions. In this context, Pam3CSK4 is a synthetic Toll-like receptor (TLR) 1/2 agonist that induces proinflammatory responses [97]. The splenic bacterial load is measurably increased in *Salmonella*-challenged neutrophils isolated from GDD knockout mice, regardless of the presence of deoxyribonuclease (DNase) [96]. The time-dependent localization of cleaved GDD with plasma membranes, as well as on released NETs, is observed using PMA-activated neutrophils [98]. However, GDD is not cleaved in PMA-stimulated neutrophils isolated from a CGD patient [98], and NET release is significantly reduced in PMA-activated neutrophils from GDD-deficient mice [98].

Treating isolated human neutrophils with an NE inhibitor reduces GDD processing, implying that NE cleaves and activates GDD [98]. Conversely, the incubation of neutrophils with a GDD inhibitor inhibited NE granule release, suggesting that GDD regulates NE activity [98]. Consequently, the NE proteolytic activation of GDD induces the further granule release of NE, promoting a potential GDD-NE positive feedback loop that supports NET formation [98]. In this context, NE translocates to the nucleus to mediate chromatin decondensation, while activated GDD localizes to the plasma membrane in order to induce membrane breakdown.

#### 2.1.5. Chromatin Release

NET release is a host defense mechanism as a response to inflammatory or infectious stimuli [99]. Decorated with neutrophil-associated granular proteins, the extracellular chromatin forms scaffold-like structures, which limit bacterial dissemination and further tissue invasion by ensnaring and neutralizing infectious pathogens [55,100,101,102]. A number of inflammatory triggers can induce NET formation, including cathepsin C, G-CSF, IL-1β, IL-8, IFN-γ, and TNF-α [103,104,105,106]. Both Gram-negative and -positive bacteria are also known to be potent inducers of NET release [107] (Table 1).

Despite the diversity of pathogens that trigger NET formation, a number of pathogen-derived virulence factors play central roles in the development of bacterial resistance to such antimicrobial NETs. Effective virulence factors are produced by several Gram-positive bacteria, such as *Streptococcus pneumoniae*, *Streptococcus suis*, *Streptococcus pyogenes,* or *Staphylococcus aureus*. *S. pneumoniae*, associated with community-wide pneumonia, expresses surface-bound endonucleases that digest extracellular chromatin and so promote the bacterial evasion of NETs [117]. *S. suis*, associated with meningitis, also secretes *S. suis* nuclease A (SsnA), a bacterial DNase, which degrades NETs to promote bacterial survival [116]. Additionally, *S. pyogenes*, associated with necrotizing fasciitis, produces M1 proteins, a virulence factor involved in epithelial cell invasion which inhibits the antimicrobial cathelicidin functions of LL-37 [118]. *S. aureus*, associated with sepsis, produces nucleases that also degrade NETs to avoid entrapment and bacterial killing [119]. Additionally, several Gram-negative bacteria, including *Pseudomonas aeruginosa* and *Haemophilus influenzae*, elaborate protective virulence factors. *P. aeruginosa*, associated with pulmonary infections and sometimes isolated from cystic fibrosis patients, can develop a mucoid-like phenotype that confers resistance to NET-mediated bacterial killing [109]. Similarly, non-typable *H. influenzae*, associated with otitis media, produces biofilm-forming lipooligosaccharides that promote NET resistance [120].

### 2.2. Regulation of NET Formation

There are at least two apparently independent mechanisms of NET production. One involves an NADPH oxidase-dependent pathway, closely associated with bursts of cytosolic ROS. The other involves calcium and potassium channel activation, which appears to be associated with mitochondrial ROS bursts.

#### 2.2.1. NADPH Oxidase-Dependent Pathway

NADPH oxidase comprises multiple phagocyte oxidase (phox) subunits (gp91phox, p22phox, p40phox, p47phox, and p67phox) and Rac2 protein [121]. The activation of this enzyme complex involves intracellular signaling pathways that (a) mediate the translocation of the cytosolic components (p40phox, p47phox, p67phox, and Rac2) to the membrane (gp91phox, p22phox), and (b) contribute to the regulation of PKC phosphorylation [121,122].

NADPH oxidase plays a significant role in the antimicrobial function of neutrophils by mediating bursts of oxidation that help to clear infectious pathogens [123]. Furthermore, NADPH oxidase-dependent pathways are involved in the formation of NETs via ROS production [124]. Neither ROS production nor NET release are observed in PMA or *S. aureus*-activated neutrophils after NADPH oxidase inhibition with diphenylene iodonium (DPI) [59]. Further stimulation of DPI-treated neutrophils with glucose oxidase (GO), generating exogenous ROS, also induces NET formation [59]. Similarly, in the presence of exogenous catalase, which decomposes hydrogen peroxide into water and oxygen, there is a significant reduction in NET formation after neutrophil treatment with PMA or GO [59]. The addition of 3-amino-1,2,4-triazole (AT), which is an endogenous catalase inhibitor, however, reverses this catalase effect, resulting in significantly increased NET release [59]. Moreover, neutrophils isolated from CGD patients, deficient in NADPH oxidase functions, do not produce NETs when activated with PMA or *S. aureus* [59]. However, NET formation is restored if isolated CGD neutrophils are stimulated with GO [59].

The upstream activation of NADPH oxidase, via the Rapidly accelerated fibrosarcoma/Mitogen-activated protein kinase kinase/Extracellular signal-regulated kinase (Raf/MEK/ERK) pathway, can induce NET formation [125]. If human neutrophils are treated with either GW5074 (Raf inhibitor), U0126 (MEK inhibitor), or an ERK peptide inhibitor, they do not form NETs following activation with either PMA or *Helicobacter pylori* [125]. Treating PMA- or *H. pylori*-activated neutrophils with staurosporine, a PKC inhibitor, also inhibits NET formation, suggesting a contributing role of PKC in the formation of NETs [125]. Similarly, NET formation is inhibited by GW5074 or U0126 in neutrophils activated by the parasite *Entamoeba histolytica* [126]. In the absence of Raf or MEK inhibitors, the phosphorylation-dependent activation of ERK can be confirmed in *E. histolytica*-activated neutrophils, but phosphorylated ERK becomes undetectable in the presence of GW5074 or U0126 [126].

MPO and NE activity during NET formation involves, at least in part, ROS production [78]. The translocation of NE to the nucleus is not observed in *C. albicans*-stimulated neutrophils, which have been isolated from CGD patients [78]. Furthermore, PMA-activated neutrophils isolated from CGD patients do not cleave endogenous histone H4, implying that ROS is somehow involved in NE release from granules [78]. Similarly, treating neutrophils with PEG-catalase inhibits NE release into cytoplasm [78]. Then, the translocation of NE to the nucleus is expected to contribute to chromatin decondensation during NET formation [60]. During this, MPO remains in the neutrophil granules, while NE is translocated to the nucleus [60]. Treating neutrophils with ROS appears to mediate the MPO dissociation from NE.

Furthermore, *S. aureus* viability is reduced in the presence of PMA-activated neutrophils, particularly when H_2_O_2_ is added, in a concentration-dependent manner [127]. However, the addition of ABAH, an MPO inhibitor, significantly increases bacterial viability [127]. Incubating PMA-activated neutrophils with luminol, a compound that reacts with oxidants such as H_2_O_2_, significantly reduces NET formation [128]. This suggests the possibility of an intragranular role for ROS during NET formation. If isoluminol is used with PMA-activated neutrophils, in the place of luminol, then this effect is less pronounced [128]. These observations imply that effective ROS-neutralizing agents need to be membrane permeable, since luminol is membrane permeable, while isoluminol is membrane impermeable [129].

#### 2.2.2. Calcium and Potassium Channel-Dependent Pathway

Ca^2+^ ionophores are known to induce NET formation via an NADPH oxidase-independent pathway [130]. Ionophores A23187 and ionomycin, produced by *Streptomyces chartreusensis* and *Streptomyces conglobatus*, respectively, are able to induce NET formation [131]. When using dihydrorhodamine (DHR)123 as a fluorescent indicator of cytosolic ROS, the decreased production of ROS is observed in neutrophils treated with A23187, compared to PMA-treated neutrophils [131]. Furthermore, A23187 or ionomycin activate NET formation, even after treatment with DPI [131]. Ordinarily, significant reduction in NET formation is observed in PMA-activated neutrophils incubated with DPI [131]. While NET formation induced by Ca^2+^ ionophores does not necessarily involve cytosolic ROS, it appears to be associated with mitochondrial ROS production [131]. For example, A23187-activated neutrophils produce significantly higher levels of mitochondrial ROS when compared to PMA-activated neutrophils [131]. In addition, treating A23187-activated neutrophils with dinitrophenol (DNP), a mitochondrial uncoupler, inhibits mitochondrial ROS production and reduces NET formation [131]. In comparison, DNP does not affect NET formation through PMA-activated neutrophils [131].

In a similar manner to Ca^2+^ ionophores, the potassium ionophore nigericin, produced by *Streptomyces hygroscopicus*, induces NET release from neutrophils [99]. Furthermore, treating nigericin-activated neutrophils with pyrocatechol, an ROS scavenger, does not prevent NET formation [99]. In addition, incubating pyrocatechol with nigericin-activated neutrophils from CGD patients also does not prevent NET release [99]. Finally, treating neutrophils with 1-Ethyl-2-benzimidazolinone (1-EBIO), a potassium channel activator, induces NET release [131].

SK3 is a small conductance calcium-activated potassium channel (SK channel), which is expressed in neutrophils, and is known to play a role in cellular apoptosis [132]. If this SK3 channel is inhibited by apamin, an SK channel inhibitor, then NET formation is significantly reduced in neutrophils treated with either A23187 or ionomycin [131]. Finally, the knockdown of the gene encoding for SK3, *KCNN3*, with an siRNA in differentiated HL-60 human neutrophils, significantly reduces NET formation following activation with either A23187 or ionomycin [131]. Together, these observations demonstrate that the calcium channel-dependent pathway of NET production is at least in part dependent on a potassium channel, which is known to be associated with apoptosis.

### 2.3. NETs and Thrombosis

Immunothrombosis embraces phenomena, such as the NET-triggered formation of intravascular thrombi, as supporting defensive components of host immune responses to pathogenic infections [133]. Neutrophils serve as a potential link between hemostasis and innate immunity due to the prothrombotic potential of NETs. The expulsion of decondensed DNA strands from neutrophils can trigger coagulation via platelet and prothrombin activation, which then further propagates thrombus formation [134]. The incubation of purified human neutrophil DNA in platelet-free plasma (PFP) or platelet-rich plasma (PRP) increases thrombin generation in a concentration-dependent manner [135]. If the NETs released from PMA-activated neutrophils are incubated with platelet-poor plasma (PPP), then thrombin production is increased, while DNase treatment significantly reduces the resulting thrombin activity [136]. In this context, the co-incubation of PPP with NETs and corn trypsin inhibitors (CTIs), a FXIIa inhibitor, also reduces thrombin production.

Thrombus composition from pancreatic tumor-bearing mice is characterized by increased levels of cell-free DNA and citrullinated H3 [137]. Furthermore, the depletion of circulating neutrophils in, or DNase treatment of, mice with pancreatic tumors reduces thrombus size and weight, implying that NET release is a contributing factor for thrombus development [137]. Similarly, microthrombi are found in renal tissues collected from mice with severe glomerulonephritis following histone injections [138]. However, the pretreatment of mice with an antihistone immunoglobulin G (IgG) attenuates these effects [138]. Furthermore, thrombus formation is increased in mice with induced deep vein thrombosis (DVT) following treatment with a mixed histone solution [139]. In this setting, the presence of stained citrullinated H3 in such thrombi is consistent with the participation of NETs. If similarly prepared mice are injected with DNase, then thrombus formation, its size, and its weight are decreased, further supporting the contributing role of NETs.

Histones also modulate thrombin activity via the downregulation of anticoagulation via the thrombomodulin (TM)/activated protein C (APC) pathway [140]. Extracellular histones incubated in PPP enhance procoagulant thrombin activity, even after exogenous addition of recombinant human TM [140]. Conversely, the incubation of histones with protein C leads to the decreased production of APC in a concentration-dependent manner, even in the presence of TM [140].

### 2.4. NETs and Coagulation

Extracellular chromatin meshwork is a suitable scaffold for the assembly of coagulation factors associated with thrombus formation. Negatively charged surfaces, such as NETs, are potential triggers of Factor XII (FXII) activation. Activated FXII is known to colocalize with NETs released from PMA-activated neutrophils [141]. In this context, co-incubating activated platelets with PMA-activated neutrophils elicits significantly higher FXII activation [141]. Moreover, treating co-incubated platelets and neutrophils with antibodies targeting histones H2A-H2B, reduces FXII activation [141]. Together, this implies that (a) FXII activation is partially dependent on its association with NETs, and (b) FXII activation is further augmented in the presence of platelets.

Along with thrombin activation, neutrophil-associated enzymes as well as the released NETs can further impact fibrin production. NE and cathepsin G are known to proteolytically cleave tissue factor pathway inhibitors (TFPIs), inhibitors of both FVIIa and FXa [142]. Consequently, one may expect that the cleavage of TFPI would tend to enhance fibrin production. This effect would not be expected in NE and cathepsin G knockout mice. Indeed, fibrin deposition at the carotid ligated injury site is significantly reduced in NE and cathepsin G double knockout mouse models [142]. Furthermore, tail bleeding times are prolonged more than two-fold in mice who are deficient in NE and cathepsin G. Similarly, if wild-type mice are treated with an exogenous mutant TFPI, which is resistant to proteolysis, then prolonged bleeding at the injury site is consistently observed [142]. Moreover, if wild-type mice are infused with H2A-H2B-blocking antibodies, then fibrin production and TFPI binding are reduced, and the vessel occlusion time is prolonged [142]. Thus, NETs promote a procoagulant state by binding secreted endogenous TFPI, and proteolytically inactivating it, via NET-associated serine proteases NE and cathepsin G.

NET-associated thrombus formation can also be triggered when IgG antibodies, purified from heparin-induced thrombocytopenia (HIT) patients, are combined with heparin in whole blood [143]. Similarly, NET release can be demonstrated following the incubation of purified human neutrophils and platelets with HIT IgG antibodies, heparin, and platelet factor 4 (PF4) [143]. However, the addition of a PAD4 inhibitor, or a P-selectin- or PSGL-1- blocking antibody abolishes this effect [143]. In addition, thrombus formation can also be induced if whole blood, which has been incubated with HIT IgG antibodies and heparin, is perfused over von Willebrand Factor (vWF) [143]. Such thrombi comprise neutrophil-associated extracellular DNA, extensive fibrin deposition, and platelet aggregates. The injection of DNase during whole blood perfusion significantly reduces extracellular DNA. Furthermore, thrombus formation is observed in murine lung tissue, following treatment with HIT IgG antibodies and heparin in transgenic mice double positive for Fcγ receptor (FcγR) IIA and human PF4, which allows murine platelets to interact with HIT IgG [143]. The addition of a PAD4 inhibitor abolishes this effect, consistent with the role of NETs in thrombus formation in this setting.

Anti-β_2_-glycoprotein I (β_2_-GPI) IgG, purified from patients with antiphospholipid syndrome, induces NET release from isolated neutrophils, while the depletion of the IgG fraction abrogates this effect [144]. PPP supplemented with neutrophils and anti-β_2_-GPI monoclonal antibodies (mAbs) has increased thrombin generation [144]. In contrast, treatment with DNase reduces this effect, consistent with the role of NETs in enhanced thrombin activity. β_2_-GPI is a cationic plasma glycoprotein that interacts with surface molecules including anionic phospholipids, GPIbα, and TLR2/4 [145]. However, the interaction of β_2_-GPI with the autoantibodies directed against it enhances thrombus formation by inducing platelet activation as well as NET release [146,147].

NET–fibrinogen interactions are significantly increased when PMA-treated neutrophils are incubated with PPP [148]. The addition of DNase reduces this effect, implying that fibrinogen colocalization with neutrophil-released NETs is at least partially dependent on extracellular chromatin.

Increased NET formation is observed following the incubation of plasma obtained from gastric cancer patients with neutrophils isolated from healthy controls [149]. Thrombin and peak fibrin generation are also significantly increased following the incubation of NET-releasing neutrophils, isolated from gastric cancer patients, with plasma obtained from healthy controls. The addition of DNase during such incubations reduces these effects.

When sepsis is induced in wild-type mice, then colocalization with NETs is observed for thrombin as well as for fibrin [150]. DNase treatment is known to attenuate NET release and thrombin generation in wild-type mice. However, reduced thrombin colocalization with NETs is observed during sepsis in mice with deficiencies of PAD4 [150]. In this case, an injection of DNase does not affect thrombin activity, due to the low levels of released NETs. Together, these observations implicate a contributing role of NETs to intravascular coagulation during inflammation and sepsis.

## 3. NETs and Platelet Functions

The extracellular decondensed chromatin strands released by neutrophils function as a web-like scaffold that can potentially affect interacting cells, including platelets. Conversely, neutrophil interactions with other cells, including platelets, can alter NET release (Table 2). Rapid NET formation is observed following the co-incubation of PMA-activated neutrophils and collagen-activated platelets [141]. GPIbα-deficient mice have reduced platelet–neutrophil interactions and NET release, suggesting that platelets contribute to neutrophil recruitment and NET release [141]. In turn, activated platelets with pseudopodia-like morphology are colocalized with chromatin networks during NET perfusion with plasma containing platelets [151]. Furthermore, the perfusion of NETs with citrate dextrose-anticoagulated whole blood produces platelet aggregates adhering to the released DNA strands [151]. In contrast, the perfusion of NETs with DNase-supplemented whole blood digested the extracellular chromatin and inhibited the formation of such platelet aggregates.

The growing body of evidence is consistent with direct prothrombotic effects of certain components of NETs. For example, a shortened lag time is observed prior to the initiation of thrombin activity, along with an increased peak in thrombin production, when PRP is incubated with NETs released from PMA-activated neutrophils, even if the PRP was treated with CTI [157]. CTI is known to inhibit the plasma activation of FXII, but does not impact platelet-mediated FXII activation [158]. Similarly, even the addition of DNase does not completely counteract the reduced lag times and enhanced thrombin activity [157]. This suggests that even some dismantled NET components, such as histones, may transiently increase the prothrombotic potential of the local blood environment.

Histones are integral components of decondensed chromatin strands and possess some antimicrobial functions, which contribute toward host immune responses to infectious bacteria [55,159]. The increased intravascular formation of platelet-rich microthrombi are observed in mice injected with extracellular histones [160]. The incubation of recombinant H3 or H4 with resuspended washed platelets triggers platelet aggregation [151]. Such histone-mediated platelet aggregation involves interactions with integrin αIIbβ_3_ or with TLRs [161,162]. Kinetic studies of platelet aggregation in murine PRP treated with added histones demonstrate that the aggregation is comparable to that produced by physiological agonists, such as adenosine diphosphate (ADP) or collagen [161]. In these circumstances, intracellular calcium levels are increased following histone incubation with PRP, consistent with αIIbβ_3_ activation. Furthermore, if in this context the αIIbβ_3_ is inhibited, then platelet aggregation is reduced [161]. Similarly, histone-induced aggregation is reduced if the platelets are from mutant mice with nonfunctional αIIbβ_3_ [161]. Interestingly, however, fibrinogen-dependent microaggregates of platelets are observed in histone-stimulated mice who are deficient in αIIbβ_3_, suggesting the presence of an alternative NET-mediated platelet activation mechanism independent of αIIbβ_3_ [161].

TLRs are transmembrane pattern recognition receptors (PRRs) involved in the innate immunity detection of microbial components such as Gram-negative bacterial LPS [163]. Platelets express functional TLRs, including TLR2 and TLR4, which play a role in histone-induced platelet responses. Increased P-selectin expression and concentration-dependent platelet aggregation is observed in isolated platelets incubated with histones [164]. However, treating these isolated platelets with either anti-TLR2 or TLR4 mAb prior to histone stimulation causes significantly reduced P-selectin expression and thrombin generation. Furthermore, preincubation with TLR2- or TLR4- blocking mAbs in CTI-treated PRP increases lag times and attenuates thrombin production levels [157]. No significant additional effects are observed using both blocking mAbs, suggesting that their effects are not additive. Interestingly, the addition of DNase with both blocking mAbs increases thrombin activity lag times, compared to DNase treatment without mAbs treatment, further supporting the contributing role of platelets in NET-mediated thrombus formation [157].

Perfusing whole blood treated with HIT IgG and heparin over vWF induces NET release and platelet deposition on extracellular DNA, while the addition of DNase or a FcγRIIa inhibitor abolishes these effects [143]. Perfusing neutrophil depleted whole blood treated with HIT IgG and heparin also demonstrates no measurable extracellular DNA release or platelet accumulation. Isolated neutrophils co-incubated with platelets induce NET release when treated with HIT IgG, heparin, and PF4, while the addition of a PAD4 inhibitor or FcγRIIa blocker abolishes this effect [143]. By itself, PF4- and heparin-treated isolated neutrophils do not bind with the HIT-like monoclonal antibody in the presence of an FcγRIIa blocker [143]. Incubating PF4- and heparin-treated isolated platelets with the same FcγRIIa blocker also blocks interactions with HIT IgG, which is consistent with the FcγRIIa-mediation of platelet and neutrophil responses during NET formation.

In septic mice, platelet aggregates are colocalized with neutrophil cells and extracellular DNA [150]. Inducing sepsis in PAD4-deficient mice, however, results in a minor but significant reduction in platelet adhesion, consistent with NET-dependent platelet responses [150].

During thrombus formation, vWF is a hemostatic mediator of platelet–endothelial and platelet–platelet interactions. This bridging molecule also promotes platelet adhesion to the structural backbone of NETs via vWF–DNA interactions. Such interactions between vWF and DNA can be observed when human plasma is incubated with NETs released from PMA-activated neutrophils [151]. Similarly, extracellular DNA colocalizes with vWF in thrombus samples from baboons after the induction of DVT [151]. Consistent with this, thrombi from DVT-induced mice have vWF bound to extracellular citrullinated H3 [140]. Furthermore, when NETs are released from PMA-activated neutrophils, then DNA-dependent binding to vWF is observed even under shear conditions, while the subsequent perfusion of DNase inhibits this effect [156]. The pretreatment of wild-type vWF with isolated DNA, prior to PRP perfusion, significantly reduces platelet adhesion to A1 domain [156]. Decreased platelet adhesion is similarly observed in mutant vWF proteins lacking the A1 domain [156]. This implies a contributing role of the A1 domain in the vWF interaction with DNA.

### 3.1. Bacteria Mediated Platelet–Neutrophil Interactions

Circulating platelets and neutrophils are the host’s first responders within the hemostatic and immune systems, respectively. They play a central role in driving the vital defense mechanisms to preserve vascular integrity or prevent the spread of infections. Under shear flow, platelets are marginalized towards the periphery of the endothelial wall [165]. This places these hemostatic mediators in an ideal position to scan for gradient changes in agonist (ADP and thrombin)-dependent stimuli or to sense biomechanical force changes, such as shear stress and substrate rigidity, which may be generated within the hemodynamic microenvironment [166,167]. Platelets are also able to interact with endothelium and with crawling leukocytes, and to rapidly respond to pathogenic or inflammatory stimuli [168]. During the host response to inflammation or infection, crosstalk between the hemostatic and immune systems mediates platelet–neutrophil interactions. The incubation of purified recombinant α-hemolysin, a *S. aureus* exotoxin, induces concentration-dependent platelet–neutrophil aggregation in heparinized human whole blood [169]. Treatment with a blocking anti-CD62P antibody significantly reduces such aggregate formation, demonstrating that platelet–neutrophil interactions are P-selectin dependent. Similarly, M1 proteins, expressed by *S. pyogenes*, induce platelet–neutrophil complexes in citrated whole blood [170]. The treatment of the blood with prostacyclin, a platelet inhibitor, or with anti-CD62P-blocking antibodies, abolishes this. Moreover, septic mice infected with *S. pyogenes* also have a post-infection time-dependent increase in platelet–neutrophil complex formation [171].

Preincubating whole blood with live *Porphyromonas gingivalis* results in increased platelet–neutrophil interactions in the presence or absence of added ADP [172]. However, the highest interaction levels are observed in the presence of ADP stimulation, even for the shortest *P. gingivalis* preincubation time of 5 min. Comparatively, extending the bacterial preincubation with whole blood, even without ADP stimulation, gradually further increases the platelet–neutrophil interactions. Together, these observations suggest that the time-dependent effects of *P. gingivalis* on platelet–neutrophil interactions comprise at least two parts—a fast and a slow component [172].

Platelet interactions with neutrophils further augment the antimicrobial effectiveness of the host immune responses. Platelet depletion reduces neutrophil recruitment in mice following inflammatory stimulation or infections with *P. aeruginosa* [173,174]. Furthermore, following platelet depletion, the platelet–neutrophil aggregates and MPO levels are reduced in mice, after the induction of acute pancreatitis [175]. Similarly, both MPO levels and ROS production are reduced in thrombocytopenic or GPVI-deficient mice, following treatment with inflammatory stimuli, further supporting platelet contributions to neutrophil tissue infiltration and antimicrobial functions [174]. Additionally, the depletion of platelets reduces ROS production from leukocytes under inflammatory conditions from transfusion-related acute lung injuries in mice [176]. Consistent with such observations, platelet–neutrophil aggregates have increased oxidative burst activity compared to individual neutrophils; this is also observed in mice with sickle cell disease [177].

The neutrophil-mediated uptake of *Aggregatibacter actinomycetemcomitans* or *P. gingivalis* is increased in PRP compared to PPP [154]. Viable *A. actinomycetemcomitans* cells are significantly reduced in PRP compared to PPP, suggesting a contributing role of platelets in neutrophil-mediated phagocytosis. Furthermore, if platelet-depleted mice are challenged with systemic *Klebsiella pneumoniae* or *P. aeruginosa,* they have increased bacterial loads and reduced survival rates [173,178].

In endotoxemic mice, following LPS injections, platelets are localized with adherent neutrophils [100]. The depletion of platelets or neutrophils significantly attenuates platelet–neutrophil aggregates. Furthermore, platelet depletion significantly reduces the released NETs in LPS-treated mice [100]. Perfusing LPS-stimulated human platelets over neutrophils induces platelet adhesion to neutrophils and NET release [100]. These effects are inhibited following the addition of a blocking LFA-1 mAb, implying that, under shear conditions, the LFA-1-mediated platelet–neutrophil interactions contribute to the intravascular release of NETs.

Altogether, rapid neutrophil mobilization is crucial for the initiation of the host immune response [179]. The subsequent adhesion of neutrophils triggers the infiltration of inflamed or infected tissue and the migration towards the target site [180]. Activated platelets provide directional cues and guide crawling neutrophils towards the inflamed vasculature [181]. The functional crosstalk synergy between platelets and neutrophils contributes to a broader, more integrated antimicrobial defense against both the bacterial infection and the inflammation-induced endothelial damage.

### 3.2. Immunothrombosis: Crosstalk between the Hemostatic and Immune Systems

During pathogenic infections, the antimicrobial function of neutrophils extends beyond the immune system. Immunothrombosis comprises a collaborative response between platelets and neutrophils, which triggers NET-induced thrombus formation in response to infectious bacteria [133]. The dynamic crosstalk between the hemostatic and immune systems is coordinated to limit pathogenic dissemination and to recruit circulating neutrophils for bacterial entrapment and clearance. Chronic inflammation from persistent ongoing infections can induce endothelial dysfunction, which then downregulates vascular protective mechanisms, potentially leading to endothelial/vascular lesions [182]. Platelets are mobilized to the damaged site by adhering to the exposed ECM. The platelet activation-associated surface expression of P-selectin, an adhesion molecule that mediates platelet–platelet interactions, stabilizes the developing aggregate. P-selectin also interacts with P-selectin glycoprotein ligand-1 (PSGL-1), a neutrophil-expressed selectin ligand, which is similarly involved with neutrophil’s adhesion to the endothelium [183]. The binding of P-selectin to PSGL-1 triggers a signaling cascade that activates integrin α_M_β_2_ (MAC-1), an adhesion receptor involved with cell–cell interactions between circulating neutrophils and endothelial cells [152]. Activated MAC-1 enables the firm adhesion of neutrophils to platelets via the platelet counterreceptor, glycoprotein GPIbα [184]. With this selectin- and integrin-dependent mechanism, platelets capture neutrophils from circulation and guide them to the inflamed or infected tissue. Such heterotypic platelet–neutrophil interactions may trigger an immunothrombotic response to identify, localize, and eliminate bacteria [185].

Pattern recognition receptors (PRRs) are constitutively expressed on the membrane surface or within the endosomal compartments of neutrophils [186]. Genes for PRRs are encoded into the germline DNA, and consequently these receptors lack the kind of diversity or antigen specificity acquired either by variable(diversity)joining (V(D)J) recombination or by somatic hypermutation [187]. Rather, PRRs function as host sensors, monitoring the extracellular or cytoplasmic environment for highly conserved exogenously expressed components from invading pathogens (pathogen-associated molecular patterns (PAMPs)) or endogenously released molecules in response to vascular injury (damage-associated molecular patterns (DAMPs)) [188]. Currently, five main classes of PRRs are known, including TLRs [189]. The TLR family comprises ten functional forms, of which human neutrophils express nine (TLR 1, 2, and 4-10), while human platelets express all ten (TLR 1-10) [190,191].

#### 3.2.1. Roles of Neutrophil Toll-like Receptors

The expression of TLRs plays a key role in the microbial robust non-self sensing, as well as in regulating the functions of neutrophils and platelets [192,193]. The pretreatment of isolated human neutrophils with LPS significantly increases the release of IL-8, a proinflammatory cytokine involved with neutrophil recruitment and the expression of CD11b, a neutrophil adhesion molecule needed for firm adhesion [194]. If these neutrophils are also incubated with a p38 mitogen-activated protein kinase (MAPK) inhibitor, then the effects of LPS are attenuated. The activation of p38 MAPK regulates cytokine gene expression via the TLR signaling pathways [195]. In this context, isolated human neutrophils, when challenged with *Helicobacter pylori*, increase TLR2 and TLR4 expression [196]. Blocking TLR activity with individual anti-TLR2, anti-TLR4, or with a mixture of both mAbs, significantly reduces IL-8 and IL-10 release by neutrophils.

Injecting mice with 2′-deoxyribo(cytidine-phosphate-guanosine) (CpG) DNA produces the increased expression of TLR9 and the increased release of IL-1β and IL-12 cytokines [197]. Unmethylated CpG dinucleotides, mimicking bacterial DNA, stimulate an inflammatory response by binding to TLR9 [198]. Reduced neutrophil accumulation is observed in the corneas of mice infected with *P. aeruginosa*, following treatment with a TLR9-targeting small interfering RNA (siRNA) [198]. The siRNA inhibits the TLR9 signaling by downregulating its messenger RNA (mRNA) levels. The siRNA treatments are also associated with the increased *P. aeruginosa* bacterial load, consistent with impaired neutrophil antimicrobial function [198]. Additionally, neutrophil recruitment to lung tissues is markedly decreased in TLR2 and TLR9 deficient mice after *Saccharopolyspora rectivirgula* challenges [199]. Signaling via TLR2 is believed to be of primary importance in this context. Recurrent *S. rectivirgula* exposure to the TLR2/TLR9 double knockout mice is also associated with the diminished production of IL-17 and TNF-α cytokines, known to affect neutrophil recruitment. Increased infection susceptibility and mortality is also observed in TLR2-deficient mice when infected with *S. pneumoniae* to induce meningitis [200]. Brain tissue from these TLR2 knockout mice have increased bacterial loads when compared to controls. Moreover, neutrophils isolated from TLR2 knockout mice have delayed phagocytosis and reduced ROS-dependent bacterial clearance, when challenged with *S. pneumoniae*, compared to neutrophils from wild-type mice [201]. Ordinarily, the addition of an NADPH oxidase inhibitor significantly reduces the bactericidal activity in wild-type mouse neutrophils. However, bactericidal activity is also attenuated for TLR2-deficient mouse neutrophils, with or without the NADPH oxidase inhibitor, consistent with TLR2 role in neutrophil-mediated bacterial clearance.

#### 3.2.2. Roles of Platelet Toll-like Receptors

Platelet function extends beyond hemostasis via TLR signaling to support the antimicrobial function of neutrophils, and to contribute to the host immune response to infectious bacteria [202]. Platelet activation is significantly decreased in LPS-stimulated platelets isolated from TLR4 knockout mice when compared to those from wild-type mice [155]. This platelet activation is comparable to that in mice with a point mutation in the *tlr4* gene, causing defective LPS signal transduction. Platelets isolated from TLR2-deficient mice do not bind to collagen, and have a reduced aggregation response following treatment with Pam3CSK4, a TLR2 agonist [153]. The adhesion of isolated human platelets to collagen under shear conditions is ordinarily increased following stimulation with Pam3CSK4 [153]. Furthermore, P-selectin expression and platelet aggregation is increased when washed platelets are treated with Pam3CSK4 [153]. Pretreatment with anti-TLR2 blocking mAbs abrogates these effects on platelet adhesion and aggregation.

Intracellular phosphoinositide 3-kinase (PI3K)/Protein Kinase B (Akt) signaling also regulates platelet function, including platelet adhesion and aggregation [203]. Pam3CSK4 incubation induces concentration-dependent Akt phosphorylation in isolated human platelets, while preincubation with a PI3K inhibitor reduces this effect [153]. Platelet pretreatment with the PI3K inhibitor also attenuates platelet adhesion to collagen and platelet aggregation, consistent with the PI3K/Akt signaling role in TLR2-mediated platelet responses.

Platelet degranulation and aggregation are observed when human PRP is challenged with *S. pneumoniae* [204]. Pretreating PRP with a TLR2-blocking mAb abolishes these effects. Furthermore, *S. pneumoniae* incubation with platelet lysates demonstrates Ras-related protein 1 (RAP1) activation. RAP1, as a downstream target of PI3K, is implicated in integrin αIIbβ_3_ activation via inside-out signaling in platelets [205]. The addition of an inhibitor of PI3K, or of integrin αIIbβ_3_, inhibits platelet aggregation in PRP. This implies that TLR2-mediated platelet responses during bacterial infection likely involve the PI3K/RAP1 signaling pathway [204].

#### 3.2.3. Platelet–Neutrophil Interactions

The formation of platelet–neutrophil aggregates is increased when whole blood is treated with Pam3CSK4 [153]. In the presence of mAbs against P-selectin, however, the platelet–neutrophil aggregates are significantly decreased. Modest but significant decreases in whole blood platelet–neutrophil aggregates are also measured in the presence of TLR2-blocking mAbs [153]. Additionally, when whole blood is challenged either with periodontopathogens (*A. actinomycetemcomitans* or *P. gingivalis*), Pam3CSK4 (TLR2 agonist), or LPS (TLR4 agonist), then the formation of platelet–neutrophil aggregates is increased when compared to controls or compared to treatments with platelet agonists, such as ADP or thrombin receptor-activating peptide 6 (TRAP-6) [154]. Treatment with a TLR2-blocking mAb siginificantly reduces platelet–neutrophil aggregates in the presence of *A. actinomycetemcomitans* or *P. gingivalis*. In these circumstances, a TLR4-blocking mAb has no significant impact. However, in the presence of both TLR2- and TLR4-blocking antibodies, the platelet–neutrophil aggregates are reduced significantly for either periodontopathogen [154]. This suggests some coordination between TLR2 and TLR4 in platelet–neutrophil interactions.

When PRP is preincubated with *A. actinomycetemcomitans* or *P. gingivalis,* then there is an increased platelet surface expression of the CD40 ligand (CD40L), a proinflammatory membrane-bound or soluble member of the TNF superfamily [206]. Individual treatments with anti-TLR2 or anti-TLR4 mAbs, or with a mixture of both blocking antibodies, significantly reduce CD40L expression in PRP. Platelet CD40L expression is also induced when PRP is stimulated with a TLR2/4 agonist, further supporting the role for TLR signaling [206]. The CD40 receptors are expressed by neutrophils, enabling direct CD40L–CD40 interactions, which mediate firm adhesion between neutrophils and platelets [207]. Additionally, activated platelets represent a major source of circulating soluble CD40L (sCD40L), a trimeric CD40L fragment that indirectly promotes platelet–neutrophil crosstalk [208]. P-selectin expression is increased when isolated human platelets are treated with recombinant sCD40L [209]. Similarly, sCD40L also upregulates the surface expression and activation of MAC-1 receptors in murine neutrophils [210]. P-selectin is translocated to the surface of activated platelets and can interact with neutrophil-expressed PSGL-1 [211]. Meanwhile, MAC-1 primarily mediates neutrophil intraluminal crawling and adhesion to GPIbα-expressing platelets [212,213]. These ligand–receptor-binding models (CD40L-CD40, P-selectin-PSGL-1, and GPIbα-MAC-1) facilitate heterotypic platelet–neutrophil interactions at sites of inflammation induced by damage or infection (Table 3).

TLR4-expressing platelets can also trigger neutrophil activation and NET release. Platelet adhesion to isolated neutrophils can be stimulated by LPS, while treatment with a TLR4-inhibitory mAb significantly decreases the platelet–neutrophil interactions [214]. Furthermore, the LPS-stimulation of platelets mediates neutrophil degranulation. This effect is not observed if the LPS treatment of platelets is in the presence of a TLR4 antagonist, or if neutrophils are treated with LPS directly in the absence of platelets [214]. The incubation of LPS-activated platelets with neutrophils results in NET release even under flow. Extracellular DNA is not measured if either platelets or neutrophils are individually treated with LPS.

When platelets and neutrophils isolated from healthy donors are co-incubated with plasma from septic patients, NET formation is triggered [214]. NET release, however, is not observed when healthy control plasma is used. In the presence of platelets pretreated with LPS (TLR4 agonist), Pam3CSK4 (TLR2 agonist), *E. coli,* or *S. aureus*, neutrophils release significantly higher levels of extracellular DNA when compared to neutrophils in the absence of pre-stimulated platelets [215]. The levels of released DNA are comparable in neutrophils, incubated with platelets pretreated with an anti-TLR2 or TLR4 mAb, to neutrophils exposed to unstimulated platelets. Similarly, inhibiting the expression of GPIb or CD18 decreases NET formation in a concentration-dependent manner, implicating these receptors in platelet-induced NETs. Furthermore, platelet-induced NET formation promotes increased bacterial capture in liver sinusoids following intravenous *E. coli* injections in mice [214]. Conversely, the depletion of platelets or neutrophils abolishes this effect. Furthermore, both the mice infected intraperitoneally with *E. coli* and the LFA-1-deficient mice have significantly higher bacterial loads in their lung tissues when compared to wild-type mice [99].

Preincubating whole blood with *P. gingivalis* for 16 min, followed by ADP stimulation, increases the labeling of neutrophil-associated DNA [172]. However, prolonging preincubation to 35 min does not significantly impact DNA labeling, suggesting an optimal duration of *P. gingivalis* preincubation to maximize the labeling of neutrophil-associated DNA. Without ADP stimulation, however, the preincubation of whole blood with *P. gingivalis* for either 16 or 35 min does not significantly affect DNA labeling. This implies a requirement for platelet activation for the release of NETs from neutrophils, in response to *P. gingivalis*.

Taken together, activated platelets can induce NET release from neutrophils via a TLR-dependent mechanism. Moreover, platelets contribute to the host’s immune response by preventing bacterial dissemination through platelet-induced NET formation, and through the recruitment of neutrophils to the infection site via platelet–neutrophil interactions.

## 4. Conclusions

Neutrophils play a central role in the host immune response to infection, utilizing multiple host defense strategies, including NET formation. NET release is part of a distinct form of cell death, observed, specifically in neutrophils. Decondensed chromatin strands are decorated with granule proteins and ejected from neutrophils. The extracellular meshwork functions to limit bacterial dissemination and suppress further tissue invasion. Subsequently, ensnared pathogens are exposed to a high concentration of localized antimicrobial proteins. NET formation is also known to regulate platelet functions during infection, suggesting potential crosstalk between hemostasis and the immune system. The extracellular DNA network provides a prothrombotic scaffold that mediates the assembly of coagulation complexes and promotes the adhesion of platelets. These processes induce the activation of prothrombin and platelets, respectively, culminating in thrombus formation. Activated platelets may also function as directional markers, guiding circulating neutrophils to inflamed or infected tissue. Heterotypic cell–cell interactions between platelets and neutrophils potentiate the responses of both effector cells, triggering platelet activation and NET production. Chronic inflammation may establish a reinforcing activation loop that propagates excessive NET release and thrombus formation via dysregulated platelet–neutrophil interactions. The goals of future studies should include the characterization of the roles of specific NET components in platelet activation and other blood cell interactions. Additionally, further investigations of mechanisms mediating the platelet–neutrophil interactions are needed to elucidate the contributing factors driving a variety of thromboinflammatory complications.

## Figures and Tables

**Table 1:** Pathogenic bacteria that are known to trigger NET formation.

Classification	Bacterial Species	References
Gram-negative bacteria	*Escherichia coli*	[108]
	*Klebsiella pneumoniae*	[60]
	*Pseudomonas aeruginosa*	[109]
	*Salmonella typhimurium*	[55]
	*Shigella flexneri*	[69]
	*Vibrio cholerae*	[110]
	*Yersinia enterocolitica*	[111]
Gram-positive bacteria	*Staphylococcus aureus*	[112]
	*Streptococcus agalactiae*	[113]
	*Streptococcus gordonii*	[114]
	*Streptococcus mutans*	[115]
	*Streptococcus pneumoniae*	[101]
	*Streptococcus pyogenes*	[81]
	*Streptococcus suis*	[116]

**Table 2:** Stimuli and platelet components involved with the formation of NETs.

Ligand	Platelet Receptor	Description	References
ADP	P2Y1 P2Y12	Weak activator of platelets that can induce P-selectin expression, which facilitates platelet–neutrophil interactions	[152]
Collagen	GPVI Integrin α_2_β_1_	Can trigger platelet activation and mediate formation of NETs	[151,153]
Fibrinogen	Integrin αIIbβ_3_	Promote platelet aggregation and interact with extracellular chromatin	[148]
LPS	TLR4	Can trigger platelet or neutrophil activation, mediate platelet–neutrophil interactions, and induce NET release	[100,154,155]
Pam3CSK4	TLR2	Can induce platelet activation, promote platelet aggregation, and mediate NET formation	[96,153,154]
Thrombin	PAR1 PAR4	Strong activator of platelets that can induce P-selectin expression, which facilitates platelet–neutrophil interactions	[150]
vWF	GPIbα Integrin αIIbβ_3_	Mediates platelet adhesion to endothelium, induce platelet aggregation, and promote platelet adhesion to extracellular DNA	[151,156]

**Table 3:** Receptors and ligands contributing to platelet–neutrophil interactions.

Platelet	Neutrophil	References
CD40L	CD40	[207]
GPIbα	MAC-1	[184]
P-selectin	PSGL-1	[183]

## Abbreviations

1-EBIO, 1-Ethyl-2-benzimidazolinone; β_2_-GPI, β_2_-glycoprotein I; AA, amino acids; ADP, adenosine diphosphate; Akt, Protein Kinase B; APC, activated protein c; AT, 3-amino-1,2,4-triazole; C/EBP, cytosine-cytosine-adenosine-adenosine-thymidine-enhancer-binding protein; CAMs, cell adhesion molecules; CD40L, CD40 ligand; CDK, cyclin-dependent kinase; CGD, chronic granulomatous disease; CpG, 2′-deoxyribo(cytidine-phosphateguanosine); CTI, corn trypsin inhibitor; CXCL, chemokine (C-X-C motif) ligand; DAMP, damage-associated molecular patterns; DHR, dihydrorhodamine; DNP, dinitrophenol; DNase, deoxyribonuclease; DPI, diphenylene iodonium; DVT, deep vein thrombosis; ECM, extracellular matrix; FcγR, Fcγ receptor; FXII, factor XII; G-CSF, granulocyte–colony-stimulating factor; GDD, Gasdermin D; GM-CSF, granulocyte–macrophage colony-stimulating factor; GMPs, granulocyte-monocyte progenitors; GO, glucose oxidase; GP, glycoprotein; GTPase, guanosine triphosphatase; H, histone; HIT, heparin-induced thrombocytopenia; HSCs, hematopoietic stem cells; ICAM, intercellular cell adhesion molecule; IgG, immunoglobulin G; IL, interleukin; IVC, inferior vena cava; JAM, junctional adhesion molecule; LFA-1, leukocyte function-associated antigen-1; LPS, lipopolysaccharide; mAbs, monoclonal antibodies; MAC-1, integrin α_M_β_2_; MAC-1, macrophage-1 antigen; MAPK, mitogen-activated protein kinase; MPOs, myeloperoxidases; MPPs, multipotent progenitors; mRNA, messenger RNA; NADPH, nicotinamide adenine dinucleotide phosphate; NE, neutrophil elastase; NETs, neutrophil extracellular traps; PAD4, peptidylarginine deiminase 4; Pam3CSK4, Pam3-Cys-Ser-Lys4; PAMP, pathogen-associated molecular patterns; PECAM, platelet endothelial cell adhesion molecule; PF4, platelet factor 4; PFP, platelet-free plasma; phox, phagocyte oxidase; PI3K, phosphoinositide 3-kinase; PKC, protein kinase C; PMA, phorbol 12-myristate 13-acetate; PPP, platelet-poor plasma; PRP, platelet-rich plasma; PRRs, pattern recognition receptors; PSGL-1, P-selectin glycoprotein ligand-1; Raf/MEK/ERK, Rapidly accelerated fibrosarcoma/Mitogen-activated protein kinase kinase/Extracellular signal-regulated kinase; RAP1, Ras-related protein 1; RhoA, Ras homolog family member A; ROS, reactive oxygen species; RUNX1, runt-related transcription factor 1; sCD40L, soluble CD40L; siRNA, small interfering RNA; SK channel, small conductance calcium-activated potassium channel; SNARE, soluble N-ethylmaleimide-sensitive factor attachment protein receptor; SsnA, *Streptococcus suis* nuclease A; TFPI, tissue factor pathway inhibitor; TLR, Toll-like receptor; TM, thrombomodulin; TNF, tumor necrosis factor; TRAP-6, thrombin receptor activating peptide 6; V(D)J, variable(diversity)joining immunoglobulin domains; vWF, von Willebrand Factor; WPBs, Weibel–Palade bodies.
